# Supplementary material for: Patterns of Intron Gain and Loss in Fungi
Source: PLoS Biol. 2004 Nov 30;2(12):e422. doi: 10.1371/journal.pbio.0020422 (PMC532390; doi:10.1371/journal.pbio.0020422)
Supplement: Table S1 — Also available at http://genes.mit.edu/NielsenEtAl/. (4.3 MB ZIP). [file pbio.0020422.st001.zip › NielsenEtAl/html/1315.html]

AN7035.1.NCU04479.1.MG10171.1.FG05245.1


```
 CLUSTAL W (1.82) Multiple Sequence Alignments - Introns Inserted


Sequence 1: NCU04479.1	402 aa
Sequence 2: MG10171.1	422 aa
Sequence 3: FG05245.1	404 aa
Sequence 4: AN7035.1	390 aa
Alignment Length: 439 aa
Number Identitical Residues: 176 aa
Alignment Score (without introns) 8782


MG10171.1 	---------~-MKPT-TSILALLLPAISARFVQETDGDHVQLHP-----EVAEAEKFHIE
NCU04479.1	---------~-MKISNASLLALLLPAASARFVEQAEQNRVMLFPDGIPEEPKSTTKYHIE
FG05245.1 	MRINKSVRA1IMKFSQASLLAACLPAVSARFIEIAEVDNVVLQP---------DELFLIE
AN7035.1  	---------~-MKLSTALVLGATATGAWSYAIPQLEQEVLELPET----HYEQQEKYLIE
          	           ** : : :*.   ..  :  :   : : : *          .   : **

MG10171.1 	LAPGETRWVTEEEKWELRR0NGQRFFDITSTPELGKTLRSQSRPAAVFPSKLSQQDAVKP
NCU04479.1	LSPGDTRWVTEDEKWELRR0NGQRFFDITDHAELG-TFNKRPYKKSVFPKKPTQKKDLEP
FG05245.1 	TAPGKTQWITEEEKWEMRR0NGQNFMDITETTTLG-SKGINAESTVTFPKKCVKQDEVAK
AN7035.1  	LSPYQTRWVTEEEKWALKL0DGVNFIDITEDRNYGLFPTLDAGSYVNYPQKMGYVDTVKG
          	 :* .*:*:**:*** ::  :* .*:***.    *      .     :*.*    . :  

MG10171.1 	LLKKLAKEPMQKHLEKFTSFHTRYYK~SEYGRQSSQWLLSEVNKTIADADAHKHGVHVQH
NCU04479.1	LLKNLSKTEMEDHLTTFTSFHTRYYK0SESGRQSSEWLLKQVRDTIKAAGADDT-VTARH
FG05245.1 	LSKKLEKKNMQANLEKLTSFHTRYFK~SDYGLQSSDWVLEKVNQIIKDAGAEDT-VFAES
AN7035.1  	LIAGLSMDNMRKDLENFTSFHTRYYK~SSSGVESAQWLYDQVSKVVRDSGADKFGATVQK
          	*   *    *. .* .:*******:* *. * :*::*: .:* . :  :.*.. .. .. 

MG10171.1 	FEHSWGQNSVIATIPGKSKSTIVVGAHQDSINLFLPSILAAPGADDDGSGIVTTLEALRV
NCU04479.1	FEHAWGQNSIIATIPGKTNATVVIGAHQDSINLWLPSVLAAPGADDDGSGTVTILEAFRV
FG05245.1 	FPHTWKQHSVIATIPGQSNSTVVIGAHQDSINLFLPSILAAPGADDDGSGSVTIMEVFRA
AN7035.1  	FDHSWGQFSIIARIPGLSKKTVVLGAHQDSINLFLPSFLGAPGADDDGSGTVTILEALRG
          	* *:* * *:** *** :: *:*:*********:***.*.********** ** :*.:* 

MG10171.1 	LLTSDELAGGKLENTVEFHWYSAEEGGLLGSQAIFSTYEKQKRDIKAMLQQDMTGFIQRT
NCU04479.1	LLQSEDIIKGNHENTIEFHWYSAEEGGLLGSQAIFTTYEKARRDVKAMLQQDMTGFVSRT
FG05245.1 	LLNSKDVVKGKAPNTIEFHWYSAEEGGLLGSQAIFKSYEETGRDVKAMLQQDMTGYVQKT
AN7035.1  	LLKSDIVAHGQSPNTIEFHWYSAEEGGMLGSQAIFSKYKKNQADVKAMLQQDMTGYVQGT
          	** *. :  *:  **:***********:*******..*::   *:**********::. *

MG10171.1 	IDAGKPESVGVIVDYVDPALTEFIKTVIDE~YCDIPYVETECGYACSDHASASKAGYPSA
NCU04479.1	LQAGEVESVGVIVDYVDPNLTDFIKKIIVE~YCDIPYVETKCGYACSDHASASKAGYPSA
FG05245.1 	LDAGQPESVGVITDFVDSGLTTFIKTVVEE0YCDIPWVETKCGYACSDHASASKAGYPSA
AN7035.1  	KHAGQKESIGVMTDFVDPALTSFLKNTIVT0YCAIPFVETKCGYACSDHTSASKYGYPSA
          	 .**: **:**:.*:**. ** *:*. :   ** **:***:********:**** *****

MG10171.1 	FVIESSFDLSDNHIHTTDDKIKFLSFDHMLQHAKMTTAFVYELASTDFK-KLENESQA1I
NCU04479.1	FVIESAFEYSDNHIHTTDDLIKYLSFDHMLQHARMTLAFAYELAFADFA-KLEKGHGD~L
FG05245.1 	FVIESAFEYSDPHIHGTDDSIKYLSFDHMLQHARMTLGLVYELGFYGFDGKSEDKWSD~L
AN7035.1  	MATESTMENSNKHIHTTDDKISYLSFDHMLEHAKLTLGFAYELAFAAL----------~-
          	:. **::: *: *** *** *.:*******:**::* .:.***.   :            

MG10171.1 	AEHELSQYNRYIELDACNSLIGTP
NCU04479.1	------------------------
FG05245.1 	------------------------
AN7035.1  	------------------------
          	
```
